# National trends in prevalence, awareness, treatment, and control of hypertension among adults in Mongolia from 4 cross-sectional surveys in 2005, 2009, 2013, and 2019

**DOI:** 10.1097/MD.0000000000030140

**Published:** 2022-08-19

**Authors:** Supa Pengpid, Karl Peltzer

**Affiliations:** a Department of Health Education and Behavioral Sciences, Faculty of Public Health, Mahidol University, Bangkok, Thailand; b Department of Public Health, Sefako Makgatho Health Sciences University, Pretoria, South Africa; c Department of Psychology, College of Medical and Health Science, Asia University, Taichung, Taiwan.

**Keywords:** hypertension care, predictors, repeat cross-sectional survey

## Abstract

This study aimed to analyze trends in the prevalence, awareness, treatment, and control of hypertension and associated factors in persons 15 years and older from 2005 to 2019 in Mongolia.

National data were analyzed from 21,342 people (≥15 years) who participated in 4 cross-sectional STEPwise Approach to NCD Risk Factor Surveillance surveys in Mongolia (2005, 2009, 2013, or 2019) and had complete blood pressure measurements. The prevalence, awareness, treatment, and control of hypertension were calculated using sociodemographic factors within each study year. Logistic regression was employed to assess the associations between sociodemographic and health factors and status of hypertension, awareness, treatment, and control by study year and pooled sample.

Trend analyzes showed that the prevalence of hypertension decreased significantly from 28.4% in 2005 to 23.2% in 2019 (*P* < .001). The prevalence of awareness among hypertensives remained unchanged, the treatment among aware decreased, and the control rate increased. In adjusted logistic regression analysis with the pooled sample, male sex (adjusted odds ratio [AOR]: 1.49, 95% confidence intervals [CI]: 1.32–1.68), older age (≥45 years) (AOR: 5.90, 95% CI: 4.90–7.10), obesity (AOR: 4.29, 95% CI: 3.77–4.88), more frequent alcohol use (≥1–2 days/week) (AOR: 1.69, 95% CI: 1.39–2.05) were positively, and higher educational level (≥12 years) (AOR: 0.77, 95% CI: 0.68–0.87) and urban residence (AOR: 0.84, 95% CI: 0.74–0.97) were negatively associated with hypertension prevalence.

The prevalence of hypertension among Mongolian adults has decreased in recent years. Levels of hypertension awareness were unchanged, treatment decreased, and control increased. Increased health promotion, detection, and treatment of hypertension in Mongolia are indicated.

## 1. Introduction

Hypertension is a major medical condition causing premature death and affecting 1.3 billion adults (30–79 years) worldwide, and two-thirds of those are living in low- and middle-income countries (LMICs).^[1]^ Among adults in 44 LMIC, 17.5% had hypertension, and 39.2% were aware, 29.9% under treatment, and 10.3% controlled their hypertension.^[2]^ Trend data from 2000 to 2010 show that awareness, treatment, and control of hypertension increased substantially in high-income countries, while this was to a much lesser degree for LMICs in terms of awareness and control and even decreased for control of hypertension.^[3]^ In LMICs in East and Southeast Asia, for example, in China (adults), the proportion of crude hypertension increased from 14.0% in 1991 to 34.1% in 2015, awareness increased from 29.4% to 43.8%, treatment increased from 19.2% to 39.2% and control increased from 3.5% in 1991 to 8.4% in 2015.^[4]^

We were unable to identify studies on trends in the prevalence of hypertension, awareness, treatment, and control in Mongolia, a lower middle-income country in East Asia. In a population-based study among adults in Mongolia in 2005, 22.2% had hypertension and 24.2% were daily smokers.^[5]^ In a population survey (≥20 years) in 2017–2018 in Ulaanbaatar, Mongolia, the prevalence of hypertension was 25.6%.^[6]^ The prevalence of obesity (≥27 kg/m^2^) increased from 26.4% in 2005 to 38.3% in 2013 in Mongolia,^[7]^ and the prevalence of low physical activity increased from 10.9% in 2005 to 27.2% in 2013 in Mongolia.^[8]^ In Mongolia in 2019, high systolic blood pressure (BP) and dietary risks made the highest contribution to rates of disability-adjusted-life-years, and cardiovascular diseases (ischemic heart disease and stroke) caused the highest mortality.^[9]^ Information on the trends of the prevalence of hypertension, awareness, treatment, and control, as well as potential risk factors among the general population, are vital for strengthening strategies in preventing and controlling hypertension.^[2]^

As previously reviewed in Pengpid and Peltzer,^[10]^ some risk factors for hypertension can include an unhealthy lifestyle, such as physical inactivity, alcohol use, tobacco, overweight/obesity, poor diet (e.g., inadequate fruit and vegetable intake), and sociodemographic factors, such as increased age, gender, and lower socioeconomic status. The purpose of the study was to assess national trends in the prevalence, awareness, treatment, and control of hypertension among people 15 years and older in Mongolia from 4 cross-sectional surveys in 2005, 2009, 2013, and 2019.

## 2. Methods

### 2.1. Study design and participants

Secondary data from 4 national and cross-sectional STEPwise Approach to NCD Risk Factor Surveillance (STEPS) surveys in Mongolia in 2005, 2009, 2013, and 2019^[11]^ with complete BP measurements were analyzed. The overall response rates were 95.0% in 2005, 95.0% in 2009, 97.4% in 2013, and 98.1% in 2019.^[12–15]^ Mongolia STEPS survey data were obtained from the “World Health Organization NCD Microdata Repository.”^[11]^ Using multistage sampling stratification, a person aged 15–64 years in 2005, 2009 and 2013, and 15–69 years in 2019 was randomly chosen per household.^[12–15]^ The inclusion criteria were from those selected at random, those who consented to participate were eligible. According to the STEPS survey protocol, in step 1 socio-behavioral data, including medical history and medication use, were assessed by questionnaire, and in step 2 data on BP and anthropometry were collected.^[11]^ Anthropometric measurements were taken using the “Somatometre-Stanley 04-116 device and electronic scales GIMA.”^[12–15]^ Prior to taking BP assessments, participants were requested to sit and rest for 15 minutes, not crossing their legs. Three systolic and diastolic BP assessments were taken per person, with 3 minutes of rest between each measurement.^[11]^ Of the 3 BP measurements using the “OMRON Model M5 automatic BP monitor”^[12–15]^; the last 2 readings were averaged.^[11]^ The Mongolian Ministry of Health Medical Ethics Committee approved the study and the participants provided their written informed consent.^[12–15]^

### 2.2. Measures

#### 2.2.1. Primary outcome measure.

Hypertension was defined as “systolic BP ≥ 140 mmHg and/or diastolic BP ≥ 90 mmHg and/or previously or current treatment with antihypertensive drugs.”^[16]^

#### 2.2.2. Secondary outcome measures.

Hypertension awareness was defined as “having ever been told to have hypertension by a doctor or other healthcare worker” among all hypertensives; in the 2005 survey the reference period for the diagnosed hypertension was only in the past 12 months; therefore, in the trend analysis on awareness only the surveys in 2009, 2013, and 2019 were included. Current hypertension treatment was defined as “respondents who took antihypertensive medication within 2 weeks of the interview among those who were aware of their hypertension status.” Control of hypertension was classified as “having a desirable BP level” (i.e., an average systolic blood pressure *<* 140 mm Hg and an average diastolic blood pressure < 90 mm Hg) among those who had hypertension or among those taking antihypertensive medication.”^[11,17]^

#### 2.2.3. Covariates.

Body mass index (BMI) was classified as “underweight (<18.5 kg/m^2^), normal weight (18.5–24.9 kg/m^2^), overweight (25.0–29.9 kg/m^2^), and obesity (≥30.0 kg/m^2^).”^[11]^ Smoking status was sourced from the item, “Do you currently smoke tobacco products, such as cigarettes, cigars, or pipes?” (Yes/No). The frequency of having at least 1 standard alcoholic drink in the last 12 months was classified as “1 = never, 2 = less than once a month or 1–3 days per month, 3 = 1–2 days per week, and 4 = 3–4 days or 5–6 days per week or daily.”^[12–15]^ Insufficient fruit/vegetable intake included <5 servings/day,^[11]^ and physical activity levels (high, moderate or low) were evaluated with the “Global Physical Activity Questionnaire.”^[18]^

Sociodemographic covariates included age, sex, education in years, residence status, and ethnic group.^[11]^

### 2.3. Statistical analysis

All statistical analyses were conducted with STATA software version 14.0 (Stata Corporation, College Station, TX, USA). The weights were applied using the inverse of the probability of participant selection and adjusted for the sex and age distribution of the study sample compared to the target population.^[12–15]^ To analyze linear trends, the study year was used as a categorical variable in logistic regression analyzes, adjusted for gender, education, age group, residence, general body weight status, alcohol, and tobacco use. Logistic regressions were used to assess the associations between sociodemographic and health risk factors and hypertension, awareness, treatment, and control by study year and pooled sample. To account for the multistage sampling and weighting of the sample Taylor linearization methods were applied. *P* values < .05 were considered significant and missing values (<1.0%) were discarded. In 2005 23 (0.7%), in 2009 43 (0.8%), in 2013 39 (0.6%), and in 2019 69 (1.0%) were excluded due to lack of complete BP measurements.

## 3. Results

### 3.1. Sample characteristics

The sample with complete BP measurement included 21,342 persons (≥15 years), 3388 in 2005, 5395 in 2009, 5978 in 2013, and 6585 in 2019. The mean age slightly increased from 38.7 years (SD = 14.2) to 41.3 years (SD = 13.7) in 2019. There was an increase in educational level, urban residence, and overweight and obesity from 2005 to 2019, and a decrease of current smoking and alcohol use from 2005 to 2019. No significant changes were found regarding sex and ethnicity. Systolic and diastolic BP significantly decreased from 2005 to 2019 (see Table 1).

**Table 1:** Sample characteristics of participants aged 15 years and older, Mongolia, 2005, 2009, 2013, and 2019.

Group	Study year				*p* for trend
	2005	2009	2013	2019	
	N = 3388	N = 5395	N = 5978	N = 6585	
	N (%)	N (%)	N (%)	N (%)	
Sex					
Male	1674 (48.3)	2217 (50.6)	2719 (49.6)	2966 (49.6)	0.482
Age group (yrs)					
15–29	1033 (45.1)	1604 (47.5)	2437 (23.8)	1473 (38.7)	<0.001
30–44	1071 (33.6)	2264 (32.1)	2039 (41.9)	2491 (33.7)	0.678
45–64 or 69*	1307 (21.3)	1570 (20.4)	1537 (34.3)	2690 (27.6)	<0.001
Education (in yrs)					
0–9	1251 (37.3)	1758 (33.0)	1613 (27.2)	1868 (24.8)	<0.001
10–11	915 (28.6)	1654 (31.8)	1899 (31.5)	1712 (25.8)	<0.001
≥12	1245 (34.1)	2026 (35.1)	2501 (41.3)	3074 (49.1)	<0.001
Region					
Urban	1702 (49.7)	2546 (49.2)	2993 (46.9)	4315 (63.1)	0.028
Ethnic group					
Khalkh	2873 (85.0)	4460 (81.9)	4823 (78.5)	5621 (84.1)	0.489
Body mass index					
Normal	1930 (62.5)	2664 (55.6)	2814 (41.9)	2583 (46.2)	<0.001
Underweight	132 (5.0)	157 (4.5)	300 (3.6)	190 (4.6)	0.856
Overweight	904 (22.3)	1704 (27.4)	1731 (34.0)	2257 (30.8)	0.018
Obesity	435 (10.2)	862 (12.5)	1013 (20.5)	1441 (18.5)	<0.001
Current smoking	987 (26.6)	1403 (27.7)	1401 (27.1)	1638 (24.2)	<0.001
Alcohol use					
Never	1084 (33.1)	2227 (42.6)	2499 (35.5)	2774 (44.0)	<0.001
≤3 d/mo	1288 (37.3)	2017 (36.2)	3328 (60.6)	3667 (52.7)	<0.001
1–2 d/wk	827 (24.1)	972 (18.0)	140 (3.1)	147 (2.2)	<0.001
≥3 d/wk	212 (5.5)	217 (4.2)	34 (0.8)	65 (1.2)	<0.001
Physical activity					
Low	320 (8.2)	436 (7.4)	1403 (22.6)	1957 (28.4)	<0.001
Moderate	697 (21.1)	573 (11.5)	1513 (24.4)	1226 (18.0)	0.364
High	2394 (70.7)	4429 (81.0)	3025 (53.0)	3356 (53.6)	<0.001
Fruits/vegetables (<5 servings/d)	2952 (85.1)	4895 (89.2)	5523 (93.8)	5105 (79.5)	<0.001
	M (SD)	M (SD)	M (SD)	M (SD)	
Systolic blood pressure	124.6 (19.0)	125.6 (18.7)	127.0 (19.3)	120.5 (17.6)	<0.001
Diastolic blood pressure	76.9 (13.0)	78.9 (12.8)	79.9 (12.4)	77.3 (11.8)	<0.001

* 15–64 years in 2005, 2009 and 2013, and 15–69 years in 2019.

### 3.2. Trends in the prevalence of hypertension

Trend analyzes showed that the prevalence of hypertension decreased significantly from 28.4% in 2005 to 23.2% in 2019 (*P* < .001). The prevalence of hypertension decreased among both men and women, rural and urban residents, all age groups, and those with higher educational levels (10 or more years) but increased from 26.6% in 2005 to 30.3% in 2019 among those with lower education (0–9 years) (see Table 2).

**Table 2:** Trends in prevalence of hypertension among adults in the Mongolia STEPS survey, 2005–2019.

Variable	Study year				*p* for trend*
	2005	2009	2013	2019	
	N = 3388	N = 5395	N = 5978	N = 6585	
	N (% 95% CI)	N (% 95% CI)	N (% 95% CI)	N (% 95% CI)	
All	**1261 (28.4, 26.1–30.7**)	**1739 (27.3, 25.0–29.7**)	**1347 (27.4, 25.6–29.3**)	**1922 (23.2, 21.8–24.6**)	**<0.001**
Sex					
Female	623 (26.9, 23.6–30.4)	930 (23.1, 20.7–25.7)	643 (24.6, 22.6–26.7)	992 (21.0, 19.4–22.8)	<0.001
Male	638 (30.0, 27.4–32.7)	809 (31.4, 28.3–34.6)	704 (30.3, 27.3–33.4)	930 (25.3, 23.3–27.5)	<0.001
Age group (yrs)					
15–29	111 (10.5, 8.6–12.6)	196 (12.3, 9.4–16.1)	192 (8.3, 7.1–9.7)	122 (8.2, 6.5–10.4)	0.036
30–44	358 (32.7, 29.3–36.3)	657 (30.4, 27.7–33.4)	427 (22.3, 19.8–25.0)	527 (21.5, 19.5–23.5)	<0.001
45–64 or 69†	792 (59.7, 56.1–63.2)	886 (57.2, 54.2–60.2)	728 (47.0, 43.7–50.3)	1273 (46.2, 43.6–48.7)	<0.001
Education (in years)					
0–9	468 (26.6, 23.6–29.9)	629 (31.0, 27.5–34.8)	384 (27.7, 24.6–30.1)	655 (30.3, 27.8–33.1)	0.003
10–11	317 (27.2, 23.7–31.0)	505 (24.8, 21.5–28.4)	405 (27.4, 24.8–30.2)	557 (25.7, 23.4–28.2)	0.019
≥12	476 (31.4, 28.2–34.7)	605 (26.0, 23.5–28.7)	558 (27.2, 24.8–30.2)	710 (18.2, 16.5–20.1)	<0.001
Region					
Rural	627 (28.7, 25.3–32.4)	952 (29.3, 26.2–32.5)	687 (28.1, 25.6–30.9)	745 (25.7, 23.1–28.5)	0.008
Urban	634 (28.0, 25.2–31.0)	787 (25.2, 22.0–28.8)	660 (26.6, 24.1–29.2)	1177 (21.7, 20.1–23.3)	<0.001
Body mass index					
Normal	514 (20.0, 18.0–22.2)	586 (18.8, 16.2, 21.8)	360 (15.9, 13.3–18.8)	406 (11.4, 10.1–12.9)	<0.001
Underweight	20 (9.3, 5.7–14.3)	16 (7.6, 3.7–15.0)	18 (10.8, 6.7–17.0)	18 (7.4, 3.8–13.7)	0.610
Overweight	443 (42.2, 38.0–46.4)	639 (35.1, 31.4–38.9)	502 (32.3, 28.5–36.3)	722 (28.1, 25.8–30.5)	<0.001
Obesity	284 (60.2, 53.8–66.3)	484 (55.1, 59.4–59.7)	454 (47.5, 44.3–50.8)	757 (49.8, 46.3–53.3)	<0.001
Current smoking					
No	845 (26.0, 23.0–29.1)	1241 (25.6, 23.1–28.2)	998 (27.3, 25.7–29.0)	1401 (21.7, 20.2–23.4)	<0.001
Yes	416 (35.1, 39.6–39.8)	498 (31.7, 28.6–35.0)	349 (27.7, 23.7–32.1)	521 (27.7, 25.0–30.5)	<0.001
Alcohol use					
Never	366 (23.4, 19.6–27.6)	678 (23.9, 21.0–27.0)	468 (23.4, 21.0, 25.8)	818 (21.9, 19.8–24.1)	0.832
≤3 d/mo	464 (28.0, 25.2–31.1)	614 (26.3, 23.7–29.1)	809 (28.9, 26.6–31.4)	1019 (23.8, 22.1–25.5)	<0.001
1–2 d/wk	326 (32.8, 29.5–36.3)	361 (35.4, 31.0–40.1)	60 (44.4, 36.5–52.7)	60 (33.0, 24.8–42.5)	<0.001
≥3 d/wk	105 (41.6, 34.8–48.9)	85 (34.8, 28.0–42.3)	10 (30.8, 13.5–55.8)	25 (26.6, 16.6–39.8)	<0.001

Key findings in bold.

* Trends were tested using multivariable logistic regression adjusted for socioeconomic and health variables.

† 15–64 years in 2005, 2009 and 2013, and 15–69 years in 2019.

### 3.3. Trends in the prevalence of awareness among hypertensive individuals and treatment among aware individuals

The prevalence of awareness among hypertensive patients increased from 49.3% in 2009 to 53.0% in 2019 but was not significant (*P* = .796). This did not differ for both sexes, age group, and educational level. However, among urban residents, the prevalence of awareness among hypertensives increased significantly from 48.7% in 2009 to 58.2% in 2019 (*P* = .036) and remained unchanged among rural residents.

The prevalence of treatment among aware significantly decreased from 44.1% in 2009 to 31.5% in 2019 (*P* = .002); this decrease was higher among women (*P* = .005) than among men (*P* = .056), and the decrease was higher among younger age groups (15–29 and 30–44 years) (*P* = .010 and *P* < .001) and those with higher education (≥12 years) (*P* = .021), but there was no significant change among the older age group (45–69 years) and those with lower education (0–9 years). The prevalence of treatment among aware significantly decreased among both rural and urban residents (see Table 3).

**Table 3:** Trends in the prevalence of awareness among hypertensives and treatment among aware among adults in the Mongolia STEPS survey, 2009–2019.

Group	Study year			*p* for trend*
	2009	2013	2019	
**Awareness among hypertensives**	N = 1739	N = 1347	N = 1922	
	N (% 95% CI)	N (% 95% CI)	N (% 95% CI)	
All	**936 (49.3, 44.5–54.0**)	**766 (58.9, 54.6–63.0**)	**1152 (53.0, 49.0–57.0**)	**0.796**
Sex				
Female	614 (63.8, 59.3–68.2)	426 (66.0, 59.4–72.1)	673 (61.0, 56.1–65.7)	0.133
Male	322 (38.9, 33.0–45.1)	340 (52.9, 48.2–57.6)	479 (46.3, 41.8–51.0)	0.101
Age group (yrs)				
15–29	52 (25.5, 17.1–36.2)	40 (23.3, 16.5–31.9)	33 (18.2, 11.8–27.0)	0.209
30–44	310 (45.0, 39.9–50.3)	209 (48.9, 42.8–55.1)	236 (43.2, 37.7–48.9)	0.160
45–64 or 69†	574 (64.7, 60.0–69.1)	517 (69.0, 63.8–73.8)	883 (67.3, 63.5–70.8)	0.751
Education (in years)				
0–9	326 (47.1, 40.1–54.3)	270 (56.6, 49.0–63.9)	340 (52.5, 46.3–58.5)	0.776
10–11	222 (50.2, 43.5–56.9)	238 (63.6, 57.4–69.3)	306 (56.2, 50.3–61.8)	0.650
≥12	390 (50.8, 44.9–56.7)	306 (56.7, 49.5–63.6)	423 (51.2, 45.4–57.0)	0.684
Region				
Rural	522 (49.7, 42.2–57.2)	403 (60.2, 54.6–65.5)	401 (45.7, 38.8–52.8)	0.113
Urban	414 (48.7, 43.7–53.8)	363 (57.2, 50.8, 63.4)	751 (58.2, 53.7–62.5)	0.036
**Treatment among aware**	N = 1378	N = 1536	N = 2026	
All	**629 (44.1, 39.8–48.4**)	**285 (20.8, 17.1–25.1**)	**721 (31.5, 28.8–34.2**)	**0.002**
Sex				
Female	452 (48.1, 43.1–53.2)	171 (20.3, 16.0–25.5)	468 (33.8, 30.6–37.2)	0.005
Male	177 (38.9, 33.4–44.7)	114 (21.5, 17.2–26.5)	253 (28.6, 25.1–32.4)	0.056
Age group (years)				
15–29	29 (23.2, 15.1–34.1)	3 (1.4, 0.4–4.5)	17 (8.4, 4.7–14.4)	0.010
30–44	180 (35.3, 30.2–40.6)	61 (11.6, 8.1–16.3)	117 (19.5, 16.0–23.5)	<0.001
45–64 or 69†	420 (59.6, 53.7–65.3)	221 (29.3, 24.2–34.9)	587 (45.4, 41.8–49.0)	0.104
Education (in yrs)				
0–9	215 (44.6, 37.5–52.0)	76 (21.3, 15.4–28.7)	240 (33.8, 29.3–38.7)	0.087
10–11	184 (46.9, 40.2–53.6)	90 (19.8, 15.3–25.4)	212 (34.3, 29.6–39.3)	0.186
≥12	230 (41.3, 34.6–48.4)	119 (21.3, 16.5–27.0)	269 (27.9, 24.4–31.8)	0.021
Region				
Rural	351 (42.9, 36.3–49.7)	144 (21.1, 16.2–27.0)	246 (32.6, 28.0–37.6)	0.021
Urban	278 (45.6, 41.1–50.3)	141 (20.5, 15.2–26.9)	475 (30.9, 27.7–34.2)	0.026

Key findings in bold.

* Trends were tested using multivariable logistic regression adjusted for socioeconomic and health variables.

† 15–64 years in 2005, 2009 and 2013, and 15–69 years in 2019.

CI = confidence interval.

### 3.4. Trends in the prevalence of hypertension control

Trend analyzes showed that the prevalence of the control rate among hypertensives increased significantly from 21.6% in 2005 to 26.5% in 2019 (*P* < .001), and the control rate among treated increased significantly from 43.6% in 2005 to 52.9% in 2016 (*P* < .001). The control rate among hypertensives and/or control rate among treated increased among both men and women, in all age groups, all educational levels and both rural and urban residents (see Table 4).

**Table 4:** Trends in prevalence of control of hypertension among adults in the Mongolia STEPS survey, 2005–2019.

Group	Study year				*p* for trend*
	2005	2009	2013	2019	
**Control rate among hypertensives**	N = 3388	N = 5395	N = 5978	N = 6585	
	N (% 95% CI)	N (% 95% CI)	N (% 95% CI)	N (% 95% CI)	
All	**261 (21.6, 18.4–25.2**)	**236 (13.3, 11.0–16.0**)	**93 (7.5, 5.4–10.2**)	**516 (26.5, 23.9–29.2**)	**<0.001**
Sex					
Female	189 (32.0, 27.4–37.0)	182 (21.4, 18.0–25.2)	63 (10.0, 7.0–14.1)	339 (34.1, 30.4–38.0)	<0.001
Male	72 (11.7, 9.1–14.8)	54 (7.5, 4.9–11.4)	30 (5.3, 3.2–8.8)	177 (20.1, 16.9–23.7)	<0.001
Age group (yrs)					
15–29	21 (18.8, 12.0–28.0)	26 (14.8, 8.3–24.9)	6 (2.7, 1.0–7.2)	35 (33.9, 24.8–44.3)	0.021
30–44	88 (25.1, 20.2–30.8)	93 (12.2, 9.2–15.9)	25 (6.6, 4.1–10.4)	122 (23.6, 19.0–27.9)	<0.001
45–64 or 69†	152 (19.8, 16.8–29.9)	117 (13.4, 10.8–16.6)	62 (8.5, 5.7–12.7)	359 (26.6, 23.7–30.0)	<0.001
Education (in yrs)					
0–9	77 (16.1, 11.8–21.7)	78 (11.4, 8.0–16.1)	24 (6.4, 3.9–10.3)	158 (25.4, 21.0–30.3)	<0.001
10–11	68 (22.3, 17.4–28.3)	66 (13.8, 10.6–17.7)	30 (6.8, 4.1–10.9)	157 (25.9, 21.8–30.5)	<0.001
≥12	116 (26.2, 22.2–30.7)	92 (14.9, 12.2–18.1)	39 (8.7, 5.6–13.3)	201 (27.9, 24.0–32.1)	<0.001
Region					
Rural	126 (20.0, 15.5–25.2)	141 (13.9, 10.6–18.2)	50 (8.2, 5.2–12.7)	165 (23.9, 19.6–28.8)	0.003
Urban	135 (23.5, 19.0–28.5)	95 (12.5, 9.6–16.0)	43 (6.5, 4.2–10.1)	351 (28.3, 25.3–31.5)	<0.001
**Control rate among treated**	N = 684	N = 713	N = 340	N = 994	
All	**261 (43.6, 40.0–47.8**)	**236 (34.2, 29.5–39.2**)	**93 (26.4, 20.5–33.4**)	**516 (52.9, 48.9–56.8**)	**<0.001**
Sex					
Female	189 (48.6, 43.5–53.6)	182 (39.6, 34.0–45.4)	63 (29.1, 21.2–38.4)	339 (56.8, 51.9–61.6)	<0.001
Male	72 (34.3, 29.3–40.2)	54 (26.8, 18.6–37.0)	30 (23.2, 15.2–33.7)	177 (48.1, 41.7–54.5)	<0.001
Age group (yrs)					
15–29	21 (68.2, 49.5–82.0)	26 (58.7, 37.7–76.9)	6 (42.2, 18.3–70.3)	35 (63.4, 49.2–75.5)	0.747
30–44	88 (53.1, 46.0–60.0)	93 (38.4, 31.6–45.6)	25 (35.3, 21.9–51.5)	122 (62.8, 55.2–69.9)	<0.001
45–64 or 69†	152 (33.2, 29.1–37.3)	117 (26.0, 21.4–31.2)	62 (23.5, 16.2–32.6)	359 (46.8, 42.4–51.2)	<0.001
Education (in yrs)					
0–9	77 (37.0, 30.2–44.5)	78 (31.7, 23.3–41.5)	24 (25.5, 17.5–35.6)	158 (52.2, 45.2–59.1)	<0.001
10–11	68 (45.0, 37.1–53.4)	66 (33.6, 27.3–40.1)	30 (23.6, 15.6–36.0)	157 (51.0, 44.0–57.9)	<0.001
≥12	116 (47.5, 42.0–53.3)	92 (37.3, 31.5–43.4)	39 (29.0, 20.0–40.0)	201 (54.8, 48.9–61.0)	<0.001
Region					
Rural	126 (43.6, 37.7–49.8)	141 (36.7, 30.0–44.0)	50 (29.3, 20.7–40.0)	165 (46.0, 39.4–52.8)	0.027
Urban	135 (43.9, 38.4–49.3)	95 (31.3, 25.1–38.3)	43 (23.0, 15.8–32.1)	351 (58.0, 53.2–62.5)	<0.001

Key findings in bold.

* Trends were tested using multivariable logistic regression adjusted for socioeconomic and health variables.

† 15–64 years in 2005, 2009 and 2013, and 15–69 years in 2019.

CI = confidence interval.

### 3.5. Associations with hypertension prevalence

In the final adjusted model with the pooled sample, male sex (adjusted odds ratio [AOR]: 1.49, 95% confidence intervals [CI]: 1.32–1.68), older age (≥45 years) (AOR: 5.90, 95% CI: 4.90–7.10), obesity (AOR: 4.29, 95% CI: 3.77–4.88), more frequent alcohol use (≥1–2 days/week) (AOR: 1.69, 95% CI: 1.39–2.05) were positively associated, and higher educational level (≥12 years) (AOR: 0.77, 95% CI: 0.68–0.87) and urban residence (AOR: 0.84, 95% CI: 0.74–0.97) were negatively associated with the prevalence of hypertension. Older age and obesity were associated with hypertension prevalence in all 4 surveys, and alcohol use was associated with hypertension prevalence in the last 3 surveys. Higher education was negatively associated with the prevalence of hypertension in 2005 and 2019 (see Table 5).

**Table 5:** Associations between sociodemographic and health factors and hypertension prevalence among adults in the Mongolia STEPS survey, 2005–2019.

Variable	Study year	Study year	Study year	Study year	Pooled
	2005	2009	2013	2019	2005–2019
	AOR (95% CI)	AOR (95% CI)	AOR (95% CI)	AOR (95% CI)	AOR (95% CI)
Sex					
Female	1 (Reference)	1 (Reference)	1 (Reference)	1 (Reference)	1 (Reference)
Male	**1.26 (1.02–1.56**)*	**1.34 (1.07–1.68**)**	**0.98 (0.81–1.19**)	**0.95 (0.81–1.11**)	**1.49 (1.32–1.68**)***
Age group (yrs)					
15–29	1 (Reference)	1 (Reference)	1 (Reference)	1 (Reference)	1 (Reference)
30–44	3.05 (2.32–4.02)***	2.19 (1.66–2.90)***	2.71 (2.20–3.35)***	1.93 (1.57–2.39)***	2.08 (1.73–2.01)***
45–64 or 69†	**8.80 (6.82–11.34**)***	**6.22 (4.93–7.84**)***	**7.57 (5.93–9.66**)***	**5.07 (4.13–6.22**)***	**5.90 (4.90–7.10**)***
Education (in yrs)					
0–9	1 (Reference)	1 (Reference)	1 (Reference)	1 (Reference)	1 (Reference)
10–11	0.86 (0.67–1.12)	0.92 (0.74–1.13)	1.05 (083–1.32)	0.88 (0.73–1.05)	0.90 (0.79–1.03)
≥12	0.78 (0.61–0.98)*	0.90 (0.72–1.12)	1.01 (0.85–1.20)	0.74 (0.62–0.89)***	0.77 (0.68–0.87)***
Region					
Rural	1 (Reference)	1 (Reference)	1 (Reference)	1 (Reference)	1 (Reference)
Urban	1.02 (0.81–1.29)	0.84 (0.66–1.08)	0.80 (0.64–0.99)*	0.89 (0.75–1.07)	0.84 (0.74–0.97)*
Ethnic group					
Other	1 (Reference)	1 (Reference)	1 (Reference)	1 (Reference)	1 (Reference)
Khalkh	1.11 (0.88–1.40)	0.80 (0.63–1.02)	1.14 (0.87–1.50)	1.05 (0.85–1.30)	0.99 (0.83–1.20)
Body mass index					
Normal	1 (Reference)	1 (Reference)	1 (Reference)	1 (Reference)	1 (Reference)
Underweight	0.61 (0.35–1.05)	0.54 (0.27–1.08)	1.03 (0.60–1.75)	0.69 (0.41–1.18)	0.71 (0.47–1.07)
Overweight	2.01 (1.63–2.47)***	1.91 (1.54–2.36)***	1.95 (1.40–2.70)***	2.00 (1.70–2.35)	2.02 (1.75–2.34)***
Obesity	**3.96 (2.98–5.26**)***	**3.67 (2.77–4.85**)***	**3.81 (2.94–4.93**)***	**5.26 (4.30–6.42**)***	**4.29 (3.77–4.88**)***
Current smoking	1.13 (0.82–1.54)	0.95 (0.77–1.18)	0.96 (0.77–1.20)	1.24 (1.03–1.50)*	0.98 (0.86–1.12)
Alcohol use					
Never	1 (Reference)	1 (Reference)	1 (Reference)	1 (Reference)	1 (Reference)
≤3 d/mo	1.03 (0.80–1.34)	1.08 (0.90–1.31)	1.12 (0.95–1.32)	1.25 (1.06–1.46)**	0.95 (0.86–1.06)
1–2 d/wk	1.20 (0.88–1.62)	**1.26 (1.04–1.53**)*	**2.28 (1.45–3.57**)***	**1.98 (1.23–3.20**)**	**1.69 (1.39–2.05**)***
≥3 d/wk	1.41 (0.97–2.04)	1.19 (0.82–1.73)	2.65 (1.28–5.49)**	2.35 (1.02–5.40)*	1.39 (1.06–1.83)*
Physical activity					
Low	1 (Reference)	1 (Reference)	1 (Reference)	1 (Reference)	1 (Reference)
Moderate	0.72 (0.51–1.01)	1.07 (0.72–1.60)	0.86 (0.68–1.10)	1.04 (0.85–1.27)	0.88 (0.75–1.05)
High	0.76 (0.56–1.04)	1.05 (0.82–1.36)	0.92 (0.74–1.14)	1.05 (0.89–1.25)	0.99 (0.87–1.13)
Fruits/vegetables (<5 servings/d)	0.97 (0.73–1.28)	1.13 (0.90–1.42)	1.06 (0.79–1.41)	0.99 (0.81–1.21)	0.94 (0.80–1.11)

Key findings in bold.

*** *P* < .001;

** *P* < .01;

* *P* < .05.

† 15–64 years in 2005, 2009 and 2013, and 15–69 years in 2019.

AOR = adjusted odds ratio; CI = confidence interval.

### 3.6. Associations with hypertension awareness

In the adjusted pooled analysis, older age (≥45 years) (AOR: 6.01, 95% CI: 4.31–8.37), obesity (AOR: 1.67, 95% CI: 1.37–2.03), frequent alcohol use (≥3 days/week) (AOR: 2.11, 95% CI: 1.26–3.52), moderate physical activity (AOR: 1.51, 95% CI: 1.20–1.59), and inadequate fruit and vegetable intake (AOR: 1.39, 95% CI: 1.06–1.84) were positively, and male sex (AOR: 0.51, 95% CI: 0.44–0.60) negatively associated with awareness among hypertensives. Furthermore, urban residence (AOR: 1.63, 95% CI: 1.17–2.28) and high physical activity (AOR: 1.58, 95% CI: 1.22–2.03) were associated with awareness among hypertensive individuals in 2019. Only female sex, older age, and obesity were associated with hypertension awareness in all 3 surveys (see Table 6).

**Table 6:** Associations between sociodemographic and health factors and hypertension awareness among adults in the Mongolia STEPS survey, 2009–2019.

Variable	Study year	Study year	Study year	Pooled
	2009	2013	2019	2009–2019
	AOR (95% CI)	AOR (95% CI)	AOR (95% CI)	AOR (95% CI)
Sex				
Female	1 (Reference)	1 (Reference)	1 (Reference)	1 (Reference)
Male	**0.49 (0.36–0.67**)***	**0.57 (0.43–076**)***	**0.49 (0.38–0.62**)***	**0.51 (0.44–0.60**)***
Age group (yrs)				
15–29	1 (Reference)	1 (Reference)	1 (Reference)	1 (Reference)
30–44	2.00 (1.20–3.34)**	2.83 (1.72–4.70)***	2.93 (1.69–5.07)***	2.47 (1.79–3.42)***
45–64 or 69†	**4.46 (2.56–7.76**)***	**6.93 (3.96–12.14**)***	**7.52 (4.36–12.98**)***	**6.01 (4.31–8.37**)***
Education (in yrs)				
0–9	1 (Reference)	1 (Reference)	1 (Reference)	1 (Reference)
10–11	1.11 (0.80–1.54)	1.48 (0.94–2.32)	1.05 (0.77–1.45)	1.17 (0.96–1.43)
≥12	0.99 (0.69–1.40)	0.93 (0.58–1.48)	1.00 (0.72–1.38)	0.93 (0.75–1.14)
Region				
Rural	1 (Reference)	1 (Reference)	1 (Reference)	1 (Reference)
Urban	0.70 (0.47–1.05)	0.87 (0.55–1.37)	1.63 (1.17–2.28)**	1.04 (0.85–1.28)
Ethnic group				
Other	1 (Reference)	1 (Reference)	1 (Reference)	1 (Reference)
Khalkh	1.41 (0.80–2.48)	1.04 (0.71–1.51)	0.86 (0.55–1.37)	1.03 (0.79–1.34)
Body mass index				
Normal	1 (Reference)	1 (Reference)	1 (Reference)	1 (Reference)
Underweight	0.90 (0.29–2.81)	1.36 (0.35–5.25)	0.34 (0.08–1.45)	0.74 (0.37–1.47)
Overweight	0.91 (0.66–1.25)	1.19 (0.79–1.78)	1.60 (1.15–2.21)**	1.19 (0.98–1.46)
Obesity	**1.88 (1.26–2.80**)**	**2.06 (1.47–2.89**)***	**1.60 (1.15–2.23**)**	**1.67 (1.37–2.03**)***
Current smoking	1.11 (0.93–1.78)	1.27 (0.85–1.89)	1.22 (0.91–1.65)	1.07 (0.89–1.29)
Alcohol use				
Never	1 (Reference)	1 (Reference)	1 (Reference)	1 (Reference)
≤3 d/mo	0.93 (0.65–1.34)	1.00 (0.66–1.52)	1.43 (1.10–1.88)**	1.23 (1.01–1.50)*
1–2 d/wk	0.66 (0.46–0.96)*	1.00 (0.52–1.92)	1.91 (1.00–3.66)	0.93 (0.68–1.26)
≥3 d/wk	1.66 (0.91–3.03)	2.01 (0.39–10.43)	3.29 (0.90–11.99)	2.11 (1.26–3.52)**
Physical activity				
Low	1 (Reference)	1 (Reference)	1 (Reference)	1 (Reference)
Moderate	1.13 (0.80–1.61)	1.23 (0.82–1.86)	1.60 (1.12–2.29)*	1.51 (1.20–1.90)***
High	0.66 (0.41–1.06)	1.00 (0.50–2.03)	1.58 (1.22–2.03)***	1.20 (0.90–1.59)
Fruits/Vegetables (<5 servings/d)	0.77 (0.41–1.46)	1.18 (0.67–2.06)	1.65 (1.20–2.27)**	1.39 (1.06–1.84)*

Key findings in bold.

*** *P* < .001;

** *P* < .01;

* *P* < .05.

† 15–64 years in 2005, 2009 and 2013, and 15–69 years in 2019.

AOR = adjusted odds ratio; CI = confidence interval.

### 3.7. Associations with hypertension treatment

In the adjusted model of the pooled sample, older age (≥45 years) (AOR: 4.55, 95% CI: 3.03–6.83), overweight (AOR: 1.23, 95% CI: 1.01–1.51) and obesity (AOR: 1.59, 95% CI: 1.25–2.01), and high physical activity (AOR: 1.27, 95% CI: 1.01–1.59) were positively, and infrequent alcohol use (≤3 days/month) (AOR: 0.79, 95% CI: 0.66–0.95) was negatively associated with treatment among those who are aware. Furthermore, insufficient fruit/vegetable intake was inversely associated with treatment among those aware in 2019. Only older age was associated with treatment among those who are aware in all 3 surveys, and obesity and nonalcohol use were associated with treatment among those who are aware in 2009 and 2019 (see Table 7).

**Table 7:** Associations between sociodemographic and health factors and hypertension treatment among adults in the Mongolia STEPS survey, 2009–2019.

Variable	Study year	Study year	Study year	Pooled
	2009	2013	2019	2009–2019
	AOR (95% CI)	AOR (95% CI)	AOR (95% CI)	AOR (95% CI)
Sex				
Female	1 (Reference)	1 (Reference)	1 (Reference)	1 (Reference)
Male	0.98 (0.66–1.44)	1.01 (0.76–1.35)	0.79 (0.6–1.04)	0.86 (0.71–1.04)
Age group (yrs)				
15–29	1 (Reference)	1 (Reference)	1 (Reference)	1 (Reference)
30–44	1.80 (1.06–3.05)*	4.09 (2.30–7.28)***	2.62 (1.41–4.88)**	1.63 (1.10–2.40)*
45–64 or 69†	**4.27 (2.23–8.14**)***	**7.53 (3.67–15.44**)***	**10.11 (5.44–18.77**)***	**4.55 (3.03–6.83**)***
Education (in yrs)				
0–9	1 (Reference)	1 (Reference)	1 (Reference)	1 (Reference)
10–11	1.22 (0.85–1.75)	1.02 (0.73–1.41)	0.89 (0.66–1.21)	0.96 (0.77–1.19)
≥12	0.95 (0.65–1.37)	0.92 (0.67–1.26)	0.77 (0.56–1.03)	0.85 (0.67–1.07)
Region				
Rural	1 (Reference)	1 (Reference)	1 (Reference)	1 (Reference)
Urban	1.11 (0.83–1.49)	0.95 (0.67–1.33)	0.93 (0.71–1.22)	1.02 (0.81–1.28)
Ethnic group				
Other	1 (Reference)	1 (Reference)	1 (Reference)	1 (Reference)
Khalkh	1.18 (0.68–2.06)	1.02 (0.74–1.20)	0.80 (0.53–1.19)	1.15 (0.87–1.58)
Body mass index				
Normal	1 (Reference)	1 (Reference)	1 (Reference)	1 (Reference)
Underweight	0.38 (0.09–1.61)	0.71 (0.28–1.82)	0.71 (0.27–1.85)	0.82 (0.38–1.77)
Overweight	0.98 (0.72–1.35)	0.94 (0.66–1.33)	1.20 (0.90–1.60)	1.23 (1.01–1.51)*
Obesity	**1.82 (1.15–2.88**)*	**1.21 (0.82–1.78**)	**1.45 (1.07–1.97**)*	**1.59 (1.25–2.01**)***
Current smoking	0.57 (0.37–0.88)*	0.76 (0.48–1.20)	1.11 (0.83–1.49)	0.90 (0.70–1.15)
Alcohol use				
Never	1 (Reference)	1 (Reference)	1 (Reference)	1 (Reference)
≤3 d/mo	0.87 (0.60–1.26)	1.26 (0.99–1.60)	0.74 (0.56–0.98)*	0.79 (0.66–0.95)*
1–2 d/wk	0.54 (0.33–0.89)*	1.25 (0.65–2.38)	0.73 (0.34–1.57)	1.27 (0.88–1.84)
≥3 d/wk	0.56 (0.28–1.13)	1.15 (0.29–4.56)	0.69 (0.15–3.17)	0.91 (0.53–1.56)
Physical activity				
Low	1 (Reference)	1 (Reference)	1 (Reference)	1 (Reference)
Moderate	1.58 (0.77–3.20)	1.07 (0.74–1.54)	1.07 (0.77–1.48)	1.12 (0.88–1.42)
High	1.55 (0.80–3.01)	1.36 (0.78–2.39)	1.14 (0.88–1.49)	1.27 (1.01–1.59)*
Fruits/vegetables (<5 servings/d)	0.88 (0.42–1.87)	0.62 (0.38–1.02)	0.68 (0.49–0.93)*	0.91 (0.69–1.21)

Key findings in bold.

*** *P* < .001;

** *P* < .01;

* *P* < .05.

† 15–64 years in 2005, 2009 and 2013, and 15–69 years in 2019.

AOR = adjusted odds ratio; CI = confidence interval.

### 3.8. Associations with hypertension control among treated

In the analysis of the pooled sample, male sex (AOR: 0.60, 95% CI: 0.46–0.80), 45 or more-year-olds (AOR: 0.38, 95% CI: 0.23–0.62), obesity (AOR: 0.46, 95% CI: 0.34–0.62), moderate alcohol use (1–2 days/week) (AOR: 0.50, 95% CI: 0.27–0.93), and insufficient fruit/vegetable intake (AOR: 0.68, 95% CI: 0.49–0.95) decreased the odds of control of hypertension among treated. Hypertension control among treated was only higher among women in 2009 and 2019 and among younger participants in 2005 and 2009 (see Table 8).

**Table 8:** Associations between sociodemographic and health factors and hypertension control among treated adults in the Mongolia STEPS survey, 2005–2019.

Variable	Study year	Study year	Study year	Study year	Pooled
	2005	2009	2013	2019	2005–2019
	AOR (95% CI)	AOR (95% CI)	AOR (95% CI)	AOR (95% CI)	AOR (95% CI)
Sex					
Female	1 (Reference)	1 (Reference)	1 (Reference)	1 (Reference)	1 (Reference)
Male	**0.67 (0.44–1.02**)	**0.42 (0.23–0.76**)**	**0.95 (0.49–1.85**)	**0.53 (0.35–0.80**)**	**0.60 (0.46–0.80**)***
Age group (yrs)					
15–29	1 (Reference)	1 (Reference)	1 (Reference)	1 (Reference)	1 (Reference)
30–44	0.55 (0.25–1.20)	0.39 (0.15–1.02)	0.57 (0.11–3.06)	1.14 (0.56–2.31)	0.62 (0.36–1.08)
45–64 or 69†	**0.24 (0.11–0.53**)***	**0.26 (0.11–0.62**)**	**0.38 (0.07–2.11**)	**0.56 (0.32–1.01**)	**0.38 (0.23–0.62**)***
Education (in yrs)					
0–9	1 (Reference)	1 (Reference)	1 (Reference)	1 (Reference)	1 (Reference)
10–11	1.18 (0.70–1.96)	1.10 (0.64–1.86)	0.91 (0.41–2.02)	0.91 (0.61–1.36)	1.01 (0.78–1.31)
≥12	1.56 (0.96–2.55)	1.52 (0.87–2.65)	1.10 (0.52–2.36)	1.16 (0.77–1.76)	1.14 (0.86–1.51
Region					
Rural	1 (Reference)	1 (Reference)	1 (Reference)	1 (Reference)	1 (Reference)
Urban	1.03 (0.73–1.45)	0.73 (0.44–1.21)	0.79 (0.39–1.56)	1.67 (1.16–2.38)**	1.20 (0.91–1.57)
Ethnic group					
Other	1 (Reference)	1 (Reference)	1 (Reference)	1 (Reference)	1 (Reference)
Khalkh	0.80 (0.53–1.21)	0.81 (0.42–1.56)	1.08 (0.39–2.99)	1.04 (0.66–1.65)	0.95 (0.67–1.45)
Body mass index					
Normal	1 (Reference)	1 (Reference)	1 (Reference)	1 (Reference)	1 (Reference)
Underweight	–	0.46 (0.08–2.78)	0.90 (0.14–5.88)	–	1.14 (0.45–2.96)
Overweight	0.78 (0.52–1.17)	0.57 (0.37–0.86)**	0.79 (0.41–1.54)	0.54 (0.34–0.87)*	0.62 (0.47–0.82)***
Obesity	**0.65 (0.40–1.08**)	**0.43 (0.26–0.72**)**	**1.09 (0.48–2.48**)	**0.29 (0.18–0.45**)***	**0.46 (0.34–0.62**)***
Current smoking	0.77 (0.51–1.16)	1.35 (0.84–2.16)	0.93 (0.47–1.85)	1.09 (0.68–1.56)	1.15 (0.84–1.40)
Alcohol use					
Never	1 (Reference)	1 (Reference)	1 (Reference)	1 (Reference)	1 (Reference)
≤3 d/mo	0.90 (0.56–1.43)	1.06 (0.64–1.74)	0.91 (0.51–1.63)	1.06 (0.76–1.46)	1.04 (0.82–1.32)
1–2 d/wk	0.76 (0.44–1.33)	0.81 (0.38–1.72)	0.04 (0.004–0.34)**	0.67 (0.20–2.20)	0.50 (0.27–0.93)*
≥3 d/wk	0.82 (0.33–2.08)	2.53 (0.64–9.99)	–	0.46 (0.12–1.72)	0.90 (0.39–2.14)
Physical activity					
Low	1 (Reference)	1 (Reference)	1 (Reference)	1 (Reference)	1 (Reference)
Moderate	0.89 (0.46–1.74)	0.99 (0.30–3.32)	1.16 (0.50–2.70)	0.80 (0.49–1.31)	0.83 (0.60–1.14)
High	1.17 (0.66–2.08)	0.88 (0.29–2.68)	1.50 (0.79–2.87)	1.01 (0.68–1.49)	0.85 (0.65–1.12)
Fruits/vegetables (<5 servings/d)	1.11 (0.69–1.80)	0.68 (0.43–1.04)	0.35 (0.12–1.04)	1.02 (0.67–1.56)	0.68 (0.49–0.95)**

Key findings in bold.

*** *P* < .001;

** *P* < .01;

* *P* < .05.

† 15–64 years in 2005, 2009 and 2013, and 15–69 years in 2019.

AOR = adjusted odds ratio; CI = confidence interval.

## 4. Discussion

Trend analyses showed for the first time from repeat surveys that the prevalence of hypertension decreased significantly from 28.4% in 2005 to 23.2% in 2019 (*P* < .001) in Mongolia. Awareness among hypertensives remained unchanged, the treatment among aware decreased, and the control rate increased. In the analysis of the pooled sample, male sex, older age (≥30 years), overweight and obesity, and more frequent alcohol use increased, and higher educational level (≥12 years) and urban residence decreased the odds of hypertension prevalence. Older age, female sex, obesity, frequent alcohol use, moderate physical activity, and inadequate fruit/vegetable consumption increased the odds of awareness among hypertensives. Older age, obesity and high physical activity were associated with treatment among aware. Male sex, 45 or more-year-olds, obesity, moderate alcohol use, and insufficient fruit/vegetable intake decreased the odds of controlling hypertension among the treated.

The found prevalence of hypertension (28.4% to 23.2%) is higher than among adults in 44 LMICs (17.5%).^[2]^ Although several previous studies in the study region found an increasing trend in the prevalence of hypertension (e.g., 14.0% in 1991 to 34.1% in 2015 in China,^[4]^ 23.0% in 1991/1994 to 42.2% in 2010/2012 in urban India,^[19]^ 32.9% in 1996 to 43.5% in 2011 in Malaysia^[20]^), this study found a decreasing trend in hypertension prevalence. Like in our study, among older adults in Malaysia, the prevalence of hypertension decreased significantly.^[17]^ Possible reasons for the decrease in hypertension in Mongolia can be attributed to better control of hypertension, a decrease in current smoking, a decrease in frequent alcohol use, and an increase in fruit and vegetable consumption, despite an increase in overweight/obesity and physical inactivity. In Mongolia, the reduction of smoking, the reduction of high BP, and the reduction of poor fruit and vegetable intake can be attributed to tobacco demand-reduction measures, the increase in tobacco excise taxes, national salt/sodium policies, the management of major noncommunicable diseases, and the “drug therapy counseling for high-risk persons.”^[21,22]^ The reduction of frequent alcohol use can be attributed to the strengthening of the national alcohol prevention and control programme in 2005,^[23]^ and the “Together for Alcohol-Free Mongolia” campaign in 2011,^[24]^ and an increase in body weight and physical inactivity can in part be attributed to no change in public awareness of diet and/or physical activity.^[21]^ Furthermore, as part of the Millennium Challenge Corporation programme (2008–2013) in Mongolia, the screening for hypertension increased,^[25]^ and a multisector Better Hearts Better Cities initiative with focus on hypertension control was implemented in Ulaanbaatar, Mongolia.^[26]^

Consistent with previous research,^[10]^ we found that older age was associated with hypertension, and higher education in 2005 and 2019 was inversely associated with hypertension, as found in a systematic review.^[27]^ We found a weak negative association between urban residence and hypertension in 2013 and in the pooled sample, while some previous results^[28]^ found a positive association between urban residence and hypertension, but similar results were found in China.^[4]^ Some reasons for this may include the increase in obesity (≥30 kg/m^2^) in rural areas (12.3%) compared to urban areas (9.4%) in Mongolia.^[29]^ Overweight/obesity increased the odds of having hypertension, which is in line with previous research.^[28,30]^ Obesity can be independently associated with hypertension, but it can also be mediated by physical inactivity and a poor diet.^[31]^ We found in line with a prior systematic review,^[32]^ that frequent alcohol use increased the odds of hypertension. Another review found that there is a linear relationship between alcohol consumption and hypertension risk.^[33]^

Contrary to some previous results, we could not find a significant association between physical inactivity,^[28]^ insufficient fruit/vegetable consumption,^[34]^ current smoking,^[35]^ and hypertension. It cannot be ruled out that participants who were aware of their hypertension status adopted positive health behaviors (stopped smoking, a healthy diet and physical activity) to control their BP.^[36]^

The prevalence of awareness among hypertensives (49.3% in 2009 to 53.0% in 2019), the prevalence of treatment among aware (44.1% in 2009 to 31.5% in 2019), and the prevalence of the control rate among hypertensives (21.6% in 2005 to 26.5% in 2019) were higher than in the 44 LMICs study (aware: 39.2%, treatment: 29.9% and control: 10.3%),^[2]^ and lower in terms of awareness (69.7%) and treatment (46.8%) but not control (24.0%) in a previous study in Ulaanbaatar in 2017–2018.^[6]^ In China,^[4]^ Iran,^[37]^ and Malaysia^[17]^ awareness, treatment and control increased significantly over time,^[4,37]^ in urban India awareness, treatment, and control rates of hypertension remained unchanged,^[19]^ and in this study awareness was unchanged, treatment decreased, and control increased. Consistent with previous findings,^[2,6]^ the older age group and being a woman were associated with awareness, and control. Women may be more likely to be aware and/or controlled than men because they have better health-seeking behavior.^[38]^ Higher education did not influence awareness, treatment and/or control, as found in the multi-country study.^[2]^

Awareness, treatment and control remain suboptimal, especially among younger age groups and men, and needs to be improved by increasing screening programmes for hypertension, including annual workplace screening for hypertension,^[6]^ public awareness campaigns on hypertension,^[38]^ standardized education, and training of primary care providers on early identification, and improved management,^[39,40]^ and expansion of the implementation of the WHO HEARTS programme.^[6]^ Further research may include a longitudinal study to identify potential predictors of hypertension awareness, treatment, and control, and implementation research to scale-up health system interventions for hypertension control in Mongolia.

The study strengths include the use of nationally representative samples and standardized STEPS methodology and measures. Some variables were evaluated by self-report, which may have biased responses, and the cross-sectional design precludes causative conclusions between the evaluated variables. For the participants 15–17 years, the classification of hypertension may have been an underestimate.^[41]^ Furthermore, hypertension was only assessed in noninstitutionalized adults. Another limitation could be the potential temporal differences in the conduct of the surveys. The definition of hypertension did not use clinical criteria but the 1 commonly used in population surveys.

In conclusion, the prevalence of hypertension among Mongolian adults has decreased in recent years. Levels of hypertension awareness were unchanged, treatment decreased, and control increased. Increased health promotion, detection, and treatment of hypertension in Mongolia are indicated.

## Acknowledgments

This paper uses data from the 2005, 2009, 2013, and 2019 Mongolia STEPS survey, implemented by the Ministry of Health and Public Health Institute with the support of the World Health Organization.

## Author contributions

All authors fulfill the criteria for authorship. SP and KP conceived and designed the research, performed statistical analysis, drafted the article, and made critical revision of the article for key intellectual content. All authors read and approved the final version of the article and have agreed to authorship and order of authorship for this article.

## References

[1] World Health Organization (WHO) Hypertension. 2021. Available at: https://www.who.int/news-room/fact-sheets/detail/hypertension. [access date 1 September, 2021].

[2] Geldsetzer P Manne-Goehler J Marcus ME. The state of hypertension care in 44 low-income and middle-income countries: a cross-sectional study of nationally representative individual-level data from 1.1 million adults. Lancet. 2019;394:652–62. 31327566 10.1016/S0140-6736(19)30955-9

[3] Mills KT Bundy JD Kelly TN. Global disparities of hypertension prevalence and control: a systematic analysis of population-based studies from 90 countries. Circulation. 2016;134:441–50. 27502908 10.1161/CIRCULATIONAHA.115.018912 PMC4979614

[4] Ma S Yang L Zhao M. Trends in hypertension prevalence, awareness, treatment and control rates among Chinese adults, 1991-2015. J Hypertens. 2021;39:740–8. 33186320 10.1097/HJH.0000000000002698

[5] Bolormaa N Narantuya L De Courten M. Dietary and lifestyle risk factors for noncommunicable disease among the Mongolian population. Asia Pac J Public Health. 2008;20(suppl):23–30. 19533858

[6] Potts H Baatarsuren U Myanganbayar M. Hypertension prevalence and control in Ulaanbaatar, Mongolia. J Clin Hypertens (Greenwich). 2020;22:103–10. 31913578 10.1111/jch.13784 PMC8029771

[7] Chimeddamba O Gearon E Brilleman SL. Increases in waist circumference independent of weight in Mongolia over the last decade: the Mongolian STEPS surveys. BMC Obes. 2017;4:19. 28491328 10.1186/s40608-017-0155-3 PMC5422882

[8] Dashzeveg D Nakamura K Seino K. Changes in the configuration and patterns of physical activity among Mongolian adults, 2005-2013. J Rural Med. 2018;13:151–9. 30546804 10.2185/jrm.2977 PMC6288722

[9] Chimed-Ochir O Delgermaa V Takahashi K. Mongolia health situation: based on the Global Burden of Disease Study 2019. BMC Public Health. 2022;22:5. 34983445 10.1186/s12889-021-12070-3 PMC8729000

[10] Pengpid S Peltzer K. Prevalence, awareness, treatment and control of hypertension among adults in Kenya: cross-sectional national population-based survey. East Mediterr Health J. 2020;26:923–32. 32896887 10.26719/emhj.20.063

[11] World Health Organization (WHO). STEPwise Approach to Surveillance (STEPS). 2020. Available at: https://www.who.int/ncds/surveillance/steps/en/. [access date 22 August 2021].

[12] World Health Organization. Mongolian STEPS Survey on the Prevalence of Noncommunicable Disease Risk Factors 2006. 2007. Available at: https://extranet.who.int/ncdsmicrodata/index.php/catalog/725/related-materials. [access date 22 August 2021].

[13] World Health Organization. Mongolian STEPS Survey on the Prevalence of Noncommunicable Disease and Injury Risk Factors—2009. Manila, Philippines: World Health Organization, Regional Office for the Western Pacific 2010. Available at: https://extranet.who.int/ncdsmicrodata/index.php/catalog/619/related-materials. [access date 5 August, 2021].

[14] Public Health Institute of the Ministry of Health and Sports, Mongolia. Third National STEPS Survey on the Prevalence of Noncommunicable Disease and Injury Risk Factors-2013. Ulaanbaatar: Public Health Institute 2014. Available at: https://extranet.who.int/ncdsmicrodata/index.php/catalog/615/related-materials. [access date 5 August, 2021].

[15] National Centre for Public Health. Fourth National STEPS Survey on Prevalence of Non-Communicable Disease and Injury Risk Factors-2019. Ulaanbaatar: National Centre for Public Health 2020. Available at: https://extranet.who.int/ncdsmicrodata/index.php/catalog/836/related-materials. [access date 5 August, 2021].

[16] Chobanian AV Bakris GL Black HR. Seventh report of the joint national committee of prevention, detection, evaluation, and treatment of high blood pressure. Hypertension. 2003;42:1206–52. 14656957 10.1161/01.HYP.0000107251.49515.c2

[17] Ho BK Omar MA Sooryanarayana R. Trends in population blood pressure and prevalence, awareness, treatment and control of hypertension among older persons: the 2006 & 2015 National Health and Morbidity Survey in Malaysia. PLoS One. 2020;15:e0238780. 32911521 10.1371/journal.pone.0238780 PMC7482969

[18] Armstrong T Bull F. Development of the World Health Organization Global Physical Activity Questionnaire (GPAQ). J Public Health. 2006;14:66–70.

[19] Roy A Praveen PA Amarchand R. Changes in hypertension prevalence, awareness, treatment and control rates over 20 years in National Capital Region of India: results from a repeat cross-sectional study. BMJ Open. 2017;7:e015639. 10.1136/bmjopen-2016-015639 PMC5734355 28706098

[20] Naing C Yeoh PN Wai VN. Hypertension in Malaysia: an analysis of trends from the national surveys 1996 to 2011. Medicine (Baltim). 2016;95:e2417. 10.1097/MD.0000000000002417 PMC4718248 26765422

[21] Second Joint Mission of the United Nations Interagency Task Force on the Prevention and Control of Noncommunicable Diseases, Mongolia. September 5–9, 2016. Geneva: World Health Organization 2017.

[22] Government of Mongolia Resolution. National Programme on the Prevention and Control of Noncommunicable Diseases. 2017. Available at: chrome-extension://efaidnbmnnnibpcajpcglclefindmkaj/viewer.html?pdfurl=https%3A%2F%2Funtobaccocontrol.org%2Fimpldb%2Fwp-content%2Fuploads%2Fmongolia_2018_annex-8_NCD_program_2017.pdf&clen=212545&chunk=true. [access date 22 August, 2021].

[23] Mongolian Parliamentary Special Commission for the Prevention of Criminal Activity. National Program on Alcohol Prevention and Control. Ulaanbaatar, Mongolia: SGK 2003.

[24] Armstrong SC Tsogtbaatar B. The dual nature of alcohol use and abuse in Mongolia: reflections through policy. Asia Pac J Public Health. 2010;22(suppl 3):209S–15S. 20566556 10.1177/1010539510372836

[25] Mongolia Compact. Millennium Challenge Corporation. Available at: https://www.mcc.gov/where-we-work/program/mongolia-compact. [access date 5 February, 22].

[26] Better Hearts Better Cities | Novartis Foundation. Available at: https://www.novartisfoundation.org/our-work/enhancing-heart-health-low-income-communities/better-hearts-better-cities. [access date 5 February 22].

[27] Leng B Jin Y Li G. Socioeconomic status and hypertension: a meta-analysis. J Hypertens. 2015;33:221–9. 25479029 10.1097/HJH.0000000000000428

[28] Bjertness MB Htet AS Meyer HE. Prevalence and determinants of hypertension in Myanmar – a nationwide cross-sectional study. BMC Public Health. 2016;16:590. 27430560 10.1186/s12889-016-3275-7 PMC4950687

[29] Otgontuya D Jr Khor GL Lye MS. Obesity among mongolian adults from urban and rural areas. Malays J Nutr. 2009;15:185–94. 22691816

[30] Chow CK Teo KK Rangarajan S. Prevalence, awareness, treatment, and control of hypertension in rural and urban communities in high-, middle-, and low-income countries. JAMA. 2013;310:959–68. 24002282 10.1001/jama.2013.184182

[31] Esteghamati A Abbasi M Alikhani S. Prevalence, awareness, treatment, and risk factors associated with hypertension in the Iranian population: the national survey of risk factors for noncommunicable diseases of Iran. Am J Hypertens. 2008;21:620–6. 18451810 10.1038/ajh.2008.154

[32] Briasoulis A Agarwal V Messerli FH. Alcohol consumption and the risk of hypertension in men and women: a systematic review and meta-analysis. J Clin Hypertens. 2012;14:792–8. 10.1111/jch.12008 PMC8108791 23126352

[33] Taylor B Irving HM Baliunas D. Alcohol and hypertension: gender differences in dose-response relationships determined through systematic review and meta-analysis. Addiction. 2009;104:1981–90. 19804464 10.1111/j.1360-0443.2009.02694.x

[34] Li B Li F Wang L. Fruit and vegetables consumption and risk of hypertension: a meta-analysis. J Clin Hypertens. 2016;18:468–76. 10.1111/jch.12777 PMC8032179 26826021

[35] Leone A. Smoking and hypertension: independent or additive effects to determining vascular damage? Curr Vasc Pharmacol. 2011;9:585–93. 21143165 10.2174/157016111796642706

[36] Hussain MA Mamun AA Reid C. Prevalence, awareness, treatment and control of hypertension in Indonesian adults aged ≥40 Years: findings from the Indonesia Family Life Survey (IFLS). PLoS One. 2016;11:e0160922. 27556532 10.1371/journal.pone.0160922 PMC4996427

[37] Esteghamati A Etemad K Koohpayehzadeh J. Awareness, treatment and control of pre-hypertension and hypertension among adults in Iran. Arch Iran Med. 2016;19:456–64. 27362238

[38] Demaio AR Otgontuya D de Courten M. Hypertension and hypertension-related disease in Mongolia; findings of a national knowledge, attitudes and practices study. BMC Public Health. 2013;13:194. 23497002 10.1186/1471-2458-13-194 PMC3600051

[39] Myanganbayar M Baatarsuren U Chen G. Hypertension knowledge, attitudes, and practices of nurses and physicians in primary care in Ulaanbaatar Mongolia. J Clin Hypertens (Greenwich). 2019;21:1202–9. 31268239 10.1111/jch.13592 PMC8030526

[40] Tripathy JP Thakur JS Jeet G. Alarmingly high prevalence of hypertension and pre-hypertension in North India-results from a large cross-sectional STEPS survey. PLoS One. 2017;12:e0188619. 29267338 10.1371/journal.pone.0188619 PMC5739392

[41] Jessen N Damasceno A Silva-Matos C. Hypertension in Mozambique: trends between 2005 and 2015. J Hypertens. 2018;36:779–84. 29210894 10.1097/HJH.0000000000001618

